# Within-host dynamics of antiviral treatment with remdesivir for SARS-CoV-2 infection

**DOI:** 10.1098/rsif.2024.0536

**Published:** 2024-11-27

**Authors:** Lea Schuh, Peter V. Markov, Ioanna Voulgaridi, Zacharoula Bogogiannidou, Varvara A. Mouchtouri, Christos Hadjichristodoulou, Nikolaos I. Stilianakis

**Affiliations:** ^1^ Joint Research Centre (JRC), European Commission, Ispra, Italy; ^2^ London School of Hygiene & Tropical Medicine, University of London, London, UK; ^3^ Laboratory of Hygiene and Epidemiology, University of Thessaly, Larissa, Greece; ^4^ Department of Biometry and Epidemiology, University of Erlangen-Nuremberg, Erlangen, Germany

**Keywords:** COVID-19, infectious disease modelling, viral kinetics, antiviral treatment, mechanistic model

## Abstract

The effectiveness of antiviral treatment with remdesivir against COVID-19 has been investigated in clinical trials suggesting earlier recovery. However, this effect seems to be rather modest. In this study, we tracked the clinical course of SARS-CoV-2 infections in 369 COVID-19 individuals across a spectrum of illness severities, including both untreated individuals and individuals who received antiviral treatment with remdesivir. Moreover, using a process-based mathematical model, we quantified and analysed the within-host infection dynamics of a total of 88 individuals, of which 69 were untreated and 19 antiviral-treated individuals. For untreated individuals, we found that those hospitalized exhibit lower levels of early immune response and higher cumulative viral loads than those who were not. For treated individuals, we found that those who died were on average hospitalized later after symptom onset than those who survived, underscoring the importance of early medical intervention for severe COVID-19. Finally, our model estimates a rather limited antiviral activity of remdesivir. Our results provide valuable insights into the clinical course of COVID-19 during antiviral treatment with remdesivir and suggest the need for alternative treatment regimens.

## 1. Introduction

Acting as an adenosine triphosphate analogue, remdesivir is meant to impede RNA-dependent RNA polymerase activity, a crucial step in viral replication. The interference with this biological mechanism is expected to impact the viral dynamics of severe acute respiratory syndrome coronavirus type 2 (SARS-CoV-2) and is the basis for the desired clinical effects of remdesivir. The effectiveness of remdesivir as an antiviral in individuals infected with SARS-CoV-2 has been investigated in clinical trials, with conflicting reports regarding its impact on important clinical outcomes such as patient mortality, duration of hospital stay or time to recovery [1–7]. Despite equivocal evidence, remdesivir has been authorized for emergency use throughout the European Union and other countries including the United States, Australia and Japan. Understanding within-host viral load dynamics during the course of infection under antiviral treatment can provide insights into the mechanisms of drug action and the relationships between the process of infection and the above-mentioned clinical outcomes [8–10]. So far, studies evaluating the effectiveness of remdesivir have predominantly focused on the clinical symptoms of individuals [1,3,5], used sparse viral load data [11–13] or worked with non-human primate animal models [14]. By coupling mechanistic modelling and viral load measurements during the course of infection, viral dynamics can be quantified at the level of the individual [15–18], providing a powerful means to evaluate the individual-specific effectiveness of COVID-19 treatments on infection dynamics [19]. Here, we present a cohort of 369 individuals with COVID-19, spanning a broad range of disease severities, including both individuals treated with the antiviral drug remdesivir and untreated individuals. Leveraging individual viral load data of 88 individuals and process-based mathematical modelling, our study investigates poorer immune responses and prolonged infections among untreated hospitalized individuals. Among treated individuals, we examined the effects of hospitalization and antiviral treatment with remdesivir on survival. Furthermore, we estimated the effectiveness of antiviral treatment and compared viral load dynamics between individuals responding and not responding to treatment. Our analysis provides a comprehensive multi-parameter profile of SARS-CoV-2 infections across different disease severities, examining the antiviral effects of remdesivir on viral load dynamics.

## 2. Results

### 2.1. Description of the study cohort

**Figure 1.**
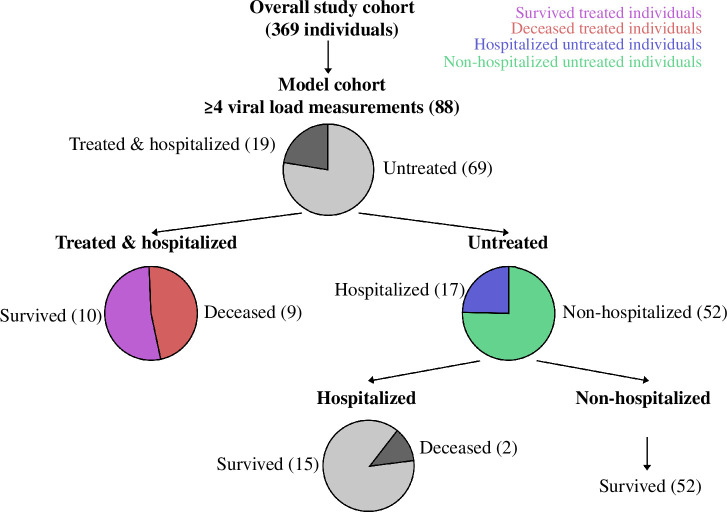
Schematic presentation of the study cohort and its subsets. Overall, 369 individuals participated in the study. Viral load measurements were taken on at least four different days for 88 individuals (model cohort). Of these, 19 individuals were treated and hospitalized. Of these in turn, 10 survived (purple) and the other 9 died from COVID-19 (red). Of the 69 untreated individuals, 17 were hospitalized (blue) and 52 were non-hospitalized (green). The proportions of the different subsets are visualized by pie charts.

Between October 2020 and September 2022, we recruited 369 individuals at the emergency department of the public hospital in Larissa, Greece, reporting symptoms consistent with COVID-19 and/or a history of close contact with a confirmed COVID-19 case and whose nasopharyngeal samples tested positive for SARS-CoV-2 (figure 1). Of these, 205 (56%) were women and 164 (44%) were men (see full cohort description in electronic supplementary material, figure S1 and supplementary information). The median age of the cohort was 49 years (range 3–99 years). Most infections were primary (96%) and less than half of the cohort was previously vaccinated (44%). For our analysis, we focused on a subset of 88 individuals for whom viral loads were measured on at least four different days through reverse transcriptase polymerase chain reaction (RT-PCR) targeting three SARS-CoV-2 specific genetic regions: ORF1ab, N and S. We refer to this subset of individuals as the ‘model cohort’, which includes 19 individuals who were hospitalized and treated with antiviral remdesivir, referred to as ‘treated individuals’ and 69 individuals who were not treated with antiviral remdesivir, referred to as ‘untreated individuals’ (electronic supplementary material, figure S2). Of the model cohort, 49 (55%) were women and 40 (45%) were men. The median age was 49 years (range 22–97 years, see electronic supplementary material, figure S1). With respect to the overall study cohort, individuals younger than 19 years of age are statistically significantly under-represented in the model cohort (*p*‐value = 0.027), while hospitalized individuals and individuals who were admitted to the ICU, intubated, and who died from COVID-19 are statistically significantly over-represented (*p*‐values = 0.012, 0.016, 0.008, and 0.025, respectively). All other parameters, such as gender, vaccination status or variant of infection, of the model cohort are representative of the overall study cohort (electronic supplementary material).

**Figure 2.**
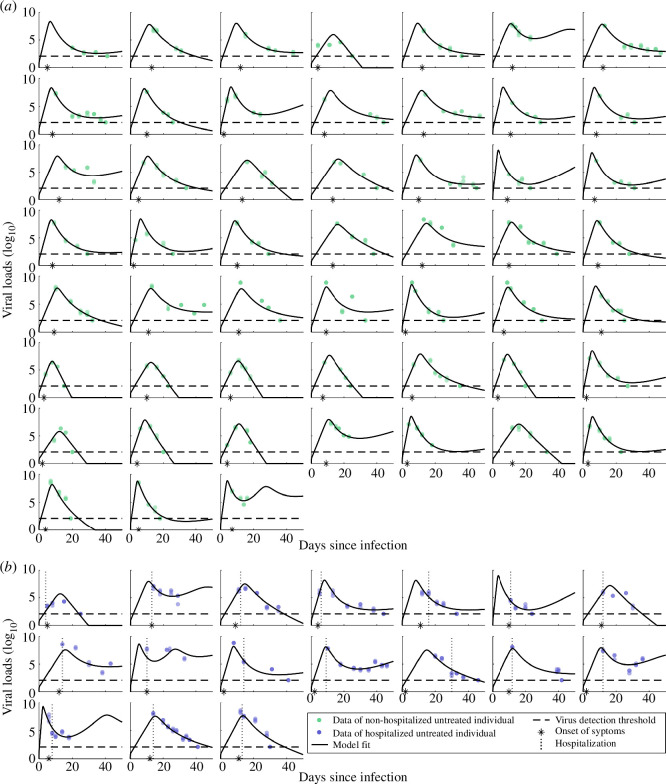
Model fits and individual-level viral load measurements of untreated individuals. (*a*) Model fits (black line) to viral loads (VL, log_10_) of 52 non-hospitalized untreated individuals (green dots). The asterisk denotes the reported day of symptom onset. The horizontal dashed line represents the detection threshold (DT) of the RT-qPCR method to detect the viral load within the sample (DT = 2.1 log_10_ VL). Dots on the dashed line denote negative test results, likely measurements below the DT. (*b*) Model fits (black line) to viral loads (log_10_) of 17 hospitalized untreated individuals (blue dots). The day of hospitalization is highlighted by a vertical dotted line. Symbols same as in (*a*).

### 2.2. Model fits to viral load dynamics in untreated individuals

To quantify the infection dynamics in untreated individuals, we fitted a within-host process-based mathematical model to viral loads derived from the observed and measured cycle threshold values for all untreated individuals (figure 2; electronic supplementary material, figures S3, S5 and table S1).

The within-host model, as previously developed in [18], describes the dynamics of susceptible and infected nasal epithelial cells, free virus and the immune response during the course of a SARS-CoV-2 infection. To capture heterogeneities between individuals, we estimated three model parameters at the individual level: the incubation period (the period from infection to onset of symptoms), the viral production rate constant and the activation rate constant of the immune response (table 1). A sensitivity analysis identified the model parameters as identifiable and robust (electronic supplementary material, figure S4). Overall, the model captures the observed viral load dynamics for both non-hospitalized and hospitalized untreated individuals (figure 2), quantifying the nuanced dynamics of mild to severe SARS-CoV-2 infections.

**Table 1.** Variables and parameter values of the model without and with treatment to describe the viral loads. Parameters with asterisks * were estimated within the given upper and lower boundaries.

variable		initial value	reference
*S*	number of susceptible cells	*S*_0_ = 8 ×10^7^	[16]
*I*	number of infected cells	*I*_0_ = 1	—
*V*	number of measured virus	*V*_0_ = 0	—
*B*	immune response	*B*_0_ = 0	—

rate constants and model parameters		value	reference
*p*_S_	susceptible cell production rate constant	*d*_S_*S*_0_ = 7.28 ×10^6^ d^−1^	—
*d*_*S*_	death rate constant of a susceptible cell	0.091 d^−1^	[20,21]
*β*_0_	infectivity rate constant	4.92 ×10^−9^ d^−1^	[16]
*d*_*I*_	death rate constant of an infected cell	2.45 d^−1^	[16]
*p*_*V*_ *	viral production rate constant	[10^2^, 10^3^] d^−1^	—
*d*_*V*_	viral clearance rate constant	10 d^−1^	[22,23]
p_B_*	activation rate constant of immune response	[10^−10^, 10^−4^] d^−1^	—
*t*_sympt_ *	incubation period (infection to symptom onset)	[2, 14] days	[24]
α*	effectiveness of remdesivir treatment	[0, 1]	—

### 2.3. Weaker early immune response and prolonged infections in hospitalized individuals

**Figure 3.**
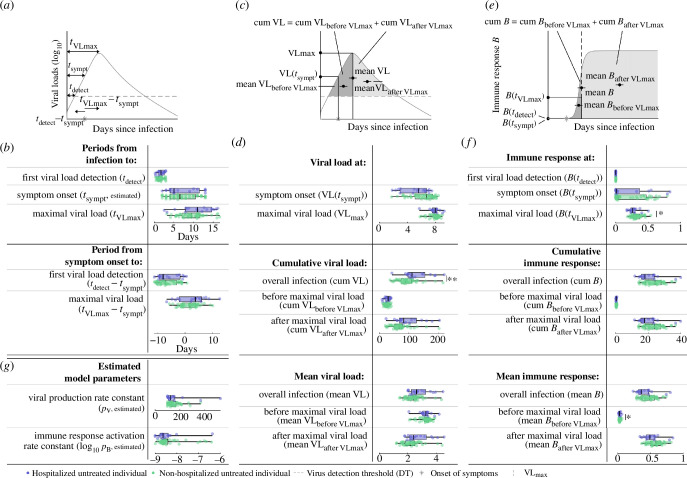
Clinical/virological timing, viral load, immune response features and model parameters for hospitalized and non-hospitalized untreated individuals. (*a*) Schematic presentation of timing features. (*b*) The estimates and predictions of the timing features in (*a*) for hospitalized (blue) and non-hospitalized (green) untreated individuals. The box plots represent the median, lower and upper quartiles, and minimum and maximum values. All features are predictions, if not specified differently. (*c*) Schematic presentation of viral load features. (*d*) The individual predictions of the viral load features from (*c*). (*e*) Schematic presentation of immune response features. (*f*) The individual predictions for the immune response features in (*e*). (*g*) Estimated rate constants. Statistically significant features are flagged with asterisks according to the level of significance.

In order to compare severe and mild COVID-19 in untreated patients, we looked at an array of dynamic features in hospitalized (severe) and non-hospitalized (mild) untreated individuals. We compared the estimated and predicted median values and distributions of timing features of the infection (figure 3*a,b*), viral loads (figure 3*c,d*) and immune response features (figure 3*e,f*), as well as the remaining estimated model parameters between these two groups (figure 3*g*) (electronic supplementary material, tables S1, S2). Specifically, the timing features are quantified by the periods

— from infection to first viral load detection,
— from infection to symptom onset,
— from infection to maximal viral load,
— from symptom onset to first viral load detection, and
— from symptom onset to maximal viral load.

The viral load features are summarized by

— the viral load at symptom onset,
— the maximal viral load,
— cumulative and mean viral loads of the overall infection,
— cumulative and mean viral loads before maximal viral load is reached, and
— cumulative and mean viral loads after maximal viral load is reached.

The immune response features are summarized by

— the immune response at first viral load detection,
— the immune response at symptom onset,
— the immune response at maximal viral load,
— the cumulative and mean immune responses of the overall infection,
— the cumulative and mean immune responses before maximal viral load is reached, and
— the cumulative and mean immune responses after maximal viral load is reached.

And the remaining estimated model parameters are

— the viral production rate constant and
— the immune response activation rate constant.

As the detection threshold (DT) of the log_10_ viral load within the sample was fixed to 2.1, it does not make sense to evaluate the viral load at detection. A summary of all tests with their respective *p*-values can be found in electronic supplementary material, table S2. The majority of the investigated features and estimated model parameters quantifying the viral load dynamics did not exhibit statistically significant differences between mild and severe COVID-19 cases. However, the predicted cumulative detectable viral load over the course of an infection, represented by the area enclosed between the predicted viral load function and the determined detection threshold, is statistically significantly greater in hospitalized untreated individuals (*p*‐value = 0.0034, figure 3*d*). Increased predicted cumulative detectable viral loads could be a result of either increased viral loads or prolonged infections. As not all individuals tested negative for SARS-CoV-2 after 50 days of predicted infection, a direct quantification and comparison of the infection duration is challenging. Instead, we quantified and compared the predicted mean viral loads during the infection course for hospitalized and non-hospitalized untreated individuals and did not find statistically significant differences between them. Overall, this suggests that hospitalized untreated individuals with severe COVID-19 probably demonstrate prolonged infections in comparison with non-hospitalized untreated individuals with mild COVID-19. Moreover, our analysis revealed a statistically significantly decreased predicted immune response at maximal viral load and a statistically significantly decreased predicted mean immune response during early infection for hospitalized untreated individuals (*p*‐values = 0.024 and 0.036, respectively, figure 3*f* and electronic supplementary material, figure S5), indicating a weaker immune response activation in this group. Interestingly, the weaker early immune response and prolonged infections in hospitalized untreated individuals with severe COVID-19 do not appear to depend solely on either the viral production rate constant or activation rate constant of the immune response (figure 3*g*).

### 2.4. Model fits to viral infection dynamics of treated individuals

**Figure 4.**
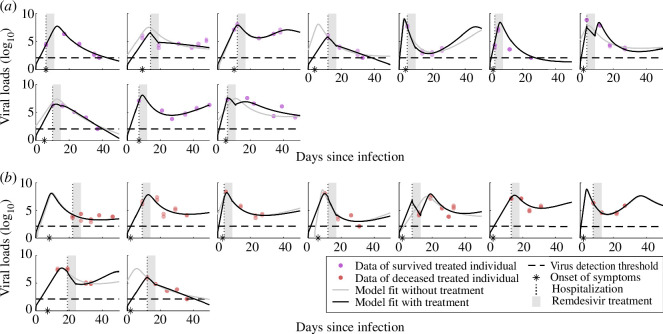
Model fits and the individual-level viral load dynamics of remdesivir-treated individuals. (*a*) Model fits with treatment (black line) and without treatment (grey line) to viral loads (log_10_) of 10 treated individuals that survived the infection (purple dots). A vertical dotted line highlights the day of hospitalization and the asterisk denotes the reported day of symptom onset. The grey area highlights the five-day period of remdesivir treatment. During that period, the viral production rate *p*_*V*_ is decreased by a factor (1 − α), where α is the proportion of reduced viral production due to treatment, termed treatment effectiveness. The horizontal dashed line represents the detection threshold (DT) of the RT-qPCR method to identify the virus in the sample (DT = 2.1 log_10_ VL). Dots on the dashed line represent negative test results, likely measurements below the DT. For some individuals, the model fits with treatment and without treatment are overlapping. (*b*) Model fits with treatment (black line) and without treatment (grey line) to viral loads (log_10_) of nine treated individuals who died from COVID-19 (red dots). Symbols same as in (*a*).

We extended the within-host model, as previously introduced in [18], to investigate the viral infection dynamics of individuals receiving antiviral treatment with remdesivir. To incorporate treatment effects into the within-host model, we allowed for a reduced viral production rate throughout the duration of treatment (electronic supplementary material, figure S3). Recognizing the variability in remdesivir’s effectiveness among individuals, we estimated it at an individual level and fitted the within-host model accounting for treatment to the viral load data of all treated individuals (figure 4; electronic supplementary material, figures S3 and S7 and table S1).

The model incorporating treatment effects described the observed viral loads for treated individuals, capturing and quantifying the dynamics of both survived and deceased treated individuals. Moreover, calculating the profile likelihoods of representative treated individuals, we found the model parameters to be robust and identifiable (electronic supplementary material, figure S6). To assess the added explanatory value of incorporating treatment effects into the model, we also fitted the model without treatment to the viral load data of the treated individuals. Quantitative evaluation using the Bayesian information criterion indicated that, in general, the model without treatment is sufficient to describe the viral load data, with only one of the 19 individuals exhibiting a considerably improved fit with the inclusion of treatment in the model (electronic supplementary material, table S3). This model comparison suggests that in most cases antiviral treatment with remdesivir may have a limited impact on viral load dynamics.

### 2.5. Early hospitalization after symptom onset is crucial for patient survival

**Figure 5.**
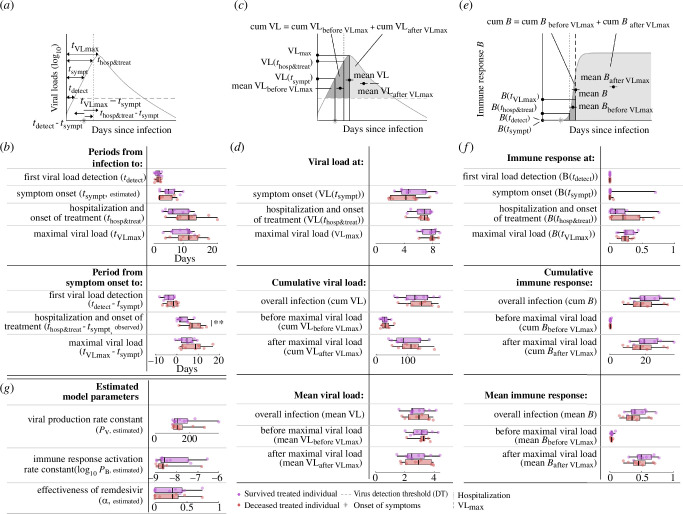
Clinical/virological timing, viral load, immune response features and model parameters for survived and deceased treated individuals. (*a*) Schematic presentation of timing features. (*b*) The observation, estimates and predictions of the timing features in (*a*) for survived (purple) and deceased (red) treated individuals. The box plots represent the median, lower and upper quartiles, and minimum and maximum values. Periods are relative to the time of symptom onset, so no box plot is shown for the incubation period. All features are predictions, if not specified differently. (*c*) Schematic presentation of viral load features. (*d*) The individual predictions of the viral load features from (*c*). (*e*) Schematic presentation of immune response features. (*f*) The individual predictions for the immune response features in (*e*). (*g*) Estimated rate constants. Statistically significant features are flagged with asterisks according to the level of significance.

Focusing on the treated individuals, we compared the median values and distributions of the previously defined dynamical features, i.e. timing (figure 5*a,b*), viral load (figure 5*c*,*d*), immune response (figure 5*e*,*f*) features and the estimated model parameters (figure 5*g*; electronic supplementary material, tables S2, S4) between the treated individuals that survived and those that died. In addition, we compared timing, viral load and immune response features related to hospitalization and treatment onset and the estimated model parameter accounting for the effectiveness of remdesivir. We could not calculate and estimate these previously for the partially non-hospitalized and untreated sub-cohort. A summary of all tests with their respective *p*-values can be found in electronic supplementary material, table S2.

Most of the investigated features and estimated model parameters quantifying the viral load dynamics do not differ statistically significantly between the survived and deceased treated individuals. However, our data suggest that deceased individuals were on average hospitalized and treated statistically significantly later after symptom onset than survived individuals (7.0 days [95% CI: 1.0–14.0 days] versus 1.5 days [95% CI: 0.0–8.0 days], respectively, with *p*‐value = 0.0021; figure 5*b*). As the time of hospitalization and the start of treatment coincide in this cohort, it is challenging to assess their relative importance. Despite the observed delay in hospitalization and the start of treatment after symptom onset in the deceased treated individuals, the predicted cumulative and mean viral loads were not statistically significantly different, indicating that viral load dynamics might not be a reliable predictor of mortality risk in this cohort (figure 5*d*). The predicted immune response features appear to be consistently smaller for deceased treated individuals, suggesting a weaker overall immune response; however, none of the differences was statistically significant. Overall, we estimated a low effectiveness of the antiviral treatment with remdesivir and found that effectiveness does not differ statistically significantly between survived and deceased treated individuals (0.3 [95% CI: 0.0–0.7%] and 0.3 [95% CI: 0.0–0.7%] respectively; figure 5*g*). Moreover, we did not find statistically significant correlations between initiation of treatment after symptom onset and the effectiveness of antiviral treatment with remdesivir in either the survived or the deceased individuals (Pearson’s correlation coefficients −0.26 (*p*‐value = 0.47) and 0.35 (*p*‐value = 0.35), respectively, using the Student’s *t*‐test).

### 2.6. Antiviral treatment with remdesivir does not substantially affect viral load dynamics

**Figure 6.**
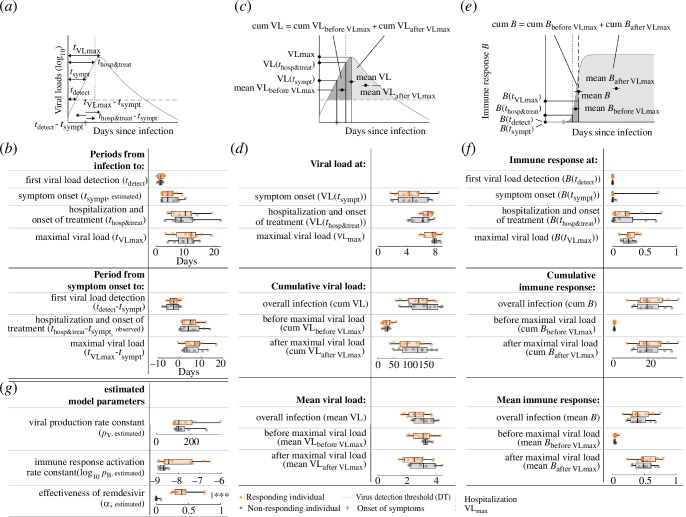
Clinical/virological timing, viral load, immune response features and model parameters for responding and non-responding individuals. (*a*) Schematic presentation of timing features. (*b*) The estimates and predictions of the timing features in (*a*) for responding (orange) and non-responding (grey) individuals. The box plots represent the median, lower and upper quartiles, and minimum and maximum values. Periods are relative to the time of symptom onset, so no box plot is shown for the incubation period. All features are predictions, if not specified differently. (*c*) Schematic presentation of viral load features. (*d*) The individual predictions of the viral load features from (*c*). (*e*) Schematic presentation of immune response features. (*f*) The individual predictions for the immune response features in (*e*). (*g*) Estimated rate constants. Statistically significant features are flagged with asterisks according to the level of significance.

Due to the non-random treatment allocation in our study cohort, comparing treated to untreated individuals to investigate the effects of antiviral treatment with remdesivir on viral load dynamics might be prone to bias or confounding. We, therefore, leveraged the estimated individual-level effectivenesses of antiviral treatment remdesivir to stratify individuals into responders (those with an estimated treatment effectiveness above 0.1) and non-responders (those with treatment effectiveness below 0.1). For this, we assumed that non-responding individuals exhibit viral load dynamics equal to untreated hospitalized individuals with comparable disease severity. We found the proportions of responders to be similar between the groups of survived (4/10) and deceased (4/9) individuals with similar median effectivenesses in both groups (0.4 and 0.3, respectively). We next compared the median values and distributions of the observed, estimated and predicted timing (figure 6*a,b*), viral load (figure 6*c,d*), immune response (figure 6*e,f*) features and estimated model parameters (figure 6*g*) between responders and non-responders (electronic supplementary material, table S2). This is the same set of comparisons as performed on the survived and deceased treated individuals. A summary of all tests with their respective *p*-values can be found in electronic supplementary material, table S2.

The effectiveness of antiviral treatment with remdesivir differed statistically significantly between responders and non-responders with median values of 0.5 and 0.0, respectively (*p*‐value = 0.000041, figure 6*g*), demonstrating an effective discriminative data split. All other features were not statistically significantly different between the two groups, suggesting that antiviral treatment with remdesivir fails to substantially reduce the viral load, influence viral load dynamics, or shorten the time to undetectable viral loads. Moreover, there is no statistically significant evidence that treatment did notably mitigate mortality between responders and non-responders, with mortalities of 50% and 45%, respectively (*p*‐value = 0.84, using the χ^2^ test). Collectively, our findings do not support a statistically significant effect of antiviral treatment with remdesivir on viral load dynamics.

## 3. Discussion

This study presents a comprehensive collection of virological, immunological and clinical characteristics of a cohort of 369 individuals infected with SARS-CoV-2. In combination with mathematical modelling, our detailed analysis of 88 individuals offers important insights into the effects of hospitalization and antiviral treatment on viral load dynamics in both untreated and remdesivir-treated individuals.

The estimated and predicted timing and viral load features, such as the period from infection to viral load detection, incubation period or the viral load at symptom onset, did not differ statistically significantly between the hospitalized individuals with severe COVID-19 and the milder non-hospitalized ones (figure 3). This result can be partially accounted for by the time discrepancy between most of the viral dynamics happening during early infection, where data collection is difficult and hence information is sparse, and the development of severe disease typically occurring later in the course of infection. It is further compatible with the well-known complex, multi-factorial determination of clinical symptoms and disease severity in COVID-19, where within-host viral dynamics is only one factor alongside the immune response and other host-specific characteristics, such as age or the presence of a range of aggravating health conditions. Viral load data were collected only after symptom onset (electronic supplementary material, figure S2), often resulting in identifiability and, hence, robustness issues of the estimated model parameters [25]. However, sensitivity analyses on the optimized model parameters identify this to be less of an issue in our study (electronic supplementary material, figures S4 and S6).

We found that the predicted cumulative viral load is statistically significantly higher in the hospitalized than in the non-hospitalized untreated individuals, while the predicted mean viral loads are not statistically significantly different between them (figure 3*d*). This parameter combination indicates longer, more protracted infections in the hospitalized untreated individuals with otherwise similar viral load levels between individuals with severe and mild COVID-19. This finding aligns with previous reports [26] and reinforces the public health recommendation for prolonged isolation for individuals suffering from moderate or severe COVID-19 [27]. Neither predicted cumulative viral loads nor predicted mean viral loads were statistically significantly different between treated individuals who died and those who survived, indicating that viral load dynamics is not a reliable predictor of mortality risk in this cohort (figure 5*d*). Our model further indicates lower predicted immune response during early infection for hospitalized untreated individuals in comparison with the non-hospitalized untreated ones (figure 3*f*). This result is compatible with both the identified longer duration of infection and the more severe disease in the group of hospitalized untreated individuals [28].

We investigated the role of antiviral treatment with remdesivir on viral dynamics using model selection and statistical approaches. We first tested if treatment as a model parameter considerably improved the model’s ability to explain the viral load data of treated individuals. The model selection did not favour the model with treatment after accounting for its additional model complexity, suggesting that antiviral treatment with remdesivir does not considerably alter viral load dynamics in our cohort. To investigate the effect of remdesivir treatment on specific key virological and clinical outcomes, while controlling for potential biases stemming from the non-random treatment allocation in our cohort, we classified treated individuals into responders and non-responders as a proxy for treated and untreated individuals (see §2 for details). In this, we assumed that non-responding individuals exhibit viral load dynamics comparable to those of untreated individuals with similar disease severity. The proportions of deceased responders and deceased non-responders are similar, suggesting that treatment does not influence mortality. Furthermore, our analysis did not find statistically significant differences in any of the investigated timing, viral load and immune response features between the groups of responders and non-responders (figure 6), suggesting that antiviral treatment with remdesivir fails to affect viral load dynamics. Finally, the median effectiveness of remdesivir between survived and deceased individuals did not differ between these two groups, also suggesting a lack of impact of remdesivir therapy administration on patient survival. Collectively, our findings do not support any statistically significant effect of antiviral treatment with remdesivir on viral load dynamics or clinical outcomes.

Our results show that the median hospitalization and onset of treatment following symptom onset was considerably delayed in individuals who eventually died, compared with those surviving the infection (figure 5*b*). This difference is substantial in magnitude and statistically significant. Survived treated individuals were hospitalized in the median of 1.5 days after the onset of their symptoms, while it took a whole week for the deceased treated individuals to be hospitalized. As treatment was initiated on the same day as hospitalization, it is challenging to disentangle the relative importance of either using this dataset. Yet, the low overall effectiveness of the antiviral treatment with remdesivir and the lack of difference between its effectiveness in survived and deceased individuals strongly points to the timing of hospitalization as the driving factor (figure 5*g*). In contrast to previous studies highlighting the importance of early antiviral administration [18,19,25], we did not find statistically significant correlations between the period from symptom onset to hospitalization/start of treatment and the effectiveness of remdesivir, underscoring the potential influence of other factors in determining treatment outcomes. Taken together, these findings suggest that early hospitalization post-symptom onset may bear greater significance for survival than antiviral treatment with remdesivir or than the timing of its initiation.

We would like to note that the longitudinal viral load data of several individuals are often described by a nonlinear mixed-effect modelling approach, which has been shown to be more robust and powerful than individual fitting [16,29]. However, having only small sample sizes per stratified class (e.g. counting only 9 and 10 individuals for deceased and survived treated, respectively) and anticipating that the overall dynamics between the classes might be similar, we decided that a nonlinear mixed effect modelling approach might fail to capture subtle differences due to its intrinsic population-based approach.

Our study suffers several limitations. We were unable to validate the model predictions for the immune response dynamics due to the lack of data. The size and scope of our sample did not permit us to stratify the data by typical confounding factors, such as age, sex, comorbidities, vaccination status, variant of infection or viral load at admission while maintaining sufficient power for statistical inference [10,30]. We took steps to minimize potential variation in sample quality collection, targeting three independent SARS-CoV-2 sub-genomic regions for RT-qPCR (N/ORF1ab/S), and then accounted for measurement noise at the modelling stage. Moreover, this study is based on a real-world cohort, where the model and sub-cohorts are only partially representative of the overall study cohort. The over-representation of hospitalized individuals within the model cohort or the large proportion of vaccinated individuals among the untreated non-hospitalized individuals may have further affected our overall results (electronic supplementary material). However, a certain inherent bias with respect to the age, hospitalization or severity in the sub-cohorts and data is expected. Finally, we would like to note that the immune response is a highly complex and dynamic process and is still poorly understood during (SARS-CoV-2) infection. The immune response is orchestrated by a variety of immune cells, including lymphocytes, phagocytes and antigen-presenting cells, which communicate and coordinate their actions through the release of cytokines, such as interleukins and interferons. These cytokines play a critical role in regulating immune cell functions, ensuring a balanced response to pathogens. There is a large variation throughout the literature on how modelling approaches incorporate the immune response and effects on the viral dynamics of SARS-CoV-2 [15,16,31,32]. In this model, the immune response is simplified and summarized by a single dynamic variable. As this model is used to describe the short-term acute infection dynamics only, we ignored a decrease in the immune response to long-term post-acute infection levels and hence a repeated increase in the overall infectivity with low viral loads.

In conclusion, our study provides important insights into SARS-CoV-2 infection dynamics in the context of antiviral treatment. Our results suggest a limited overall effectiveness of antiviral treatment with remdesivir on viral load dynamics and key clinical outcomes while demonstrating the importance of early hospitalization for survival from COVID-19. Future work might focus on using similar procedures to evaluate the clinical effectivenesses of other antiviral drugs, such as nirmatrelvir or molnupiravir, and in particular combination drug therapeutic approaches, such as paxlovid [33,34]. With minimal need for adaptation, our modelling approach is amenable to future applications, with the important potential to provide insights into COVID-19 treatment strategies and the development of resistance that would not be possible to obtain using standard epidemiological approaches [20,33,34].

## 4. Methods

### 4.1. Study design

The 369 participants were individuals who tested positive for SARS-CoV-2 between October 2020 and September 2022. These individuals sought medical assistance at a public hospital emergency department situated in Larissa, Greece, reporting symptoms consistent with COVID-19 or close contact with a confirmed case of COVID-19. All of the participants had tested positive for SARS-CoV-2 based on nasopharyngeal/oropharyngeal samples. The gender (female/male) and age of each individual were recorded (electronic supplementary material, figure S1). Moreover, we determined the specific variant of infection (alpha, beta, delta or omicron), the status of COVID-19 vaccination (yes/no), previous COVID-19 infection(s) (yes/no), emerging symptoms (yes/no), comorbidity (yes/no), hospitalization (yes/no), treatment (yes/no), admission to the intensive care unit (ICU) (yes/no), intubation (yes/no) and death from COVID-19 (yes/no). The cycle threshold (CT) was determined on at least 2 and up to 10 different days during the course of each infection using RT-PCR. The CT value denotes the number of PCR cycles required for the amplified viral load in the sample to cross the detection threshold (DT at CT = 37) and is hence inversely proportional to the viral load in the sample. We used the CT value as a proxy for the viral load. Individuals were treated with antiviral treatment remdesivir according to the therapeutic protocols for COVID-19 of the National Public Health Organization of Greece. Antiviral treatment of hospitalized individuals was recommended for individuals with the need for oxygen therapy and mild COVID-19 or with the need for high-flow oxygen therapy and/or non-invasive mechanical ventilation and/or severe COVID-19. Antiviral treatment was not recommended for individuals with clinical presentation lasting for more than 7 days, mild symptomatology and no need for oxygen therapy and for individuals in extracorporeal membrane oxygenation or mechanical ventilation except for individuals to whom the administration of antiviral treatment had already been started.

### 4.2. Laboratory analysis

Respiratory samples (nasopharyngeal or oropharyngeal swabs) were collected in transfer tubes containing viral transport medium and were analysed in the Laboratory of Hygiene and Epidemiology, Faculty of Medicine, University of Thessaly. SARS-CoV-2 RNA was isolated from the samples with KingFisher Flex System (ThermoFisher Scientific, Waltham, MA, USA) using the MagMAX™ Viral/Pathogen Nucleic Acid Isolation Kit (Applied Biosystems™, Waltham, MA, USA) according to the manufacturer’s instructions. Detection of the virus’s genetic material was performed using RT-qPCR with primers targeting SARS-CoV-2 specific genetic regions: ORF1ab, N and S, with the TaqPath™ COVID‐19 CE‐IVD RT‐PCR Kit (Applied Biosystems™, Waltham, MA, USA) on a validated QuantStudio™ 5 Real-Time PCR System (ThermoFisher Scientific, Waltham, MA, USA). The threshold for positivity less than or equal to 37 CT value for SARS-CoV-2 infection was established.

### 4.3. Model cohort and pre-processing of individual viral load data

We focused our analysis on the subset of 90 individuals for which CT value measurements were taken for at least four time points during the SARS-CoV-2 infection. We extracted their personal ID numbers (in the format VOCXXX), measurement dates and corresponding CT values, date of symptom onset, whether the individuals were treated with antivirals (yes/no), were hospitalized (yes/no), and if so, the date of hospitalization, and, finally, whether the individuals died from COVID-19. We manually standardized the reported dates to the common date format MM/DD/YYYY. We next converted the dates at which the CT values were measured and if applicable, hospitalization, to days relative to the recorded individual-specific day of symptom onset (electronic supplementary material, figure S2). Missing CT values per target gene (N/ORF/S), denoted by --, were not accounted for. Negative results, denoted by NEG(-), were only reported if, and only if, all three target gene replicates were reported below the detection threshold of 37. For negative results, we set the CT values of all three replicates to the detection threshold. We discarded one individual for whom the dates of CT value measurements were not recorded in a subsequent order and another individual who was treated but not hospitalized. In total, we considered 88 of the 369 individuals for the modelling analysis (figure 1).

### 4.4. Representativeness of subsets

To identify the features that are statistically significantly over- or under-represented in the subset of the model cohort with respect to the overall study cohort and the subsets of hospitalized and non-hospitalized untreated individuals and survived and deceased treated individuals with respect to the model cohort, we performed a statistical analysis (electronic supplementary material, figure S1A and supplementary information). For each feature and each subset, we simulated 10 000 sample subsets of X sample individuals, where X is the sample size of the respective subset, using MATLAB function *binornd*(X*,p,10000,1)* [35]. Here, *p* is the success probability given by the fraction of the feature within the larger cohort (electronic supplementary material, figure S1B). From the resulting 10 000 simulated sample subsets each of size X for a given feature, we calculated a point estimate and the 95% confidence interval. If the recorded fraction of the given feature of the subset is contained within the confidence interval, the feature is well represented by the subset. If the fraction of the given feature of the subset is below/above the confidence interval, the feature is under-/over-represented by the subset. As an example, in the overall study cohort 56% of the recorded individuals were women. To identify whether the 55% of women within the model cohort are representative of the overall study cohort, we simulated 10 000 sample subsets of 88 individuals with a success probability of *p* = 0.56. The resulting confidence interval with a confidence level of 95% is [45%, 66%]. As the recorded 55% of women in the model cohort is contained within the confidence interval, we term the fraction of women in the model cohort as representative of the fraction of women in the overall study cohort. The corresponding *p*-values were determined by using the MATLAB function *cdf* (‘Normal’,Y,mean(Y),std(Y)) [36], where Y is the simulated sample subset of X. The null hypothesis is that the recorded fraction of the sub-cohort is drawn from the simulated sample subset Y at a significance level of 0.05.

### 4.5. Within-host SARS-CoV-2 model without treatment

The model, as previously proposed in [18], comprises four populations corresponding to susceptible nasal epithelial cells *S*, infected nasal epithelial cells *I*, free virus particles *V* and the immune response *B* (normalized to values between 0 and 1) (electronic supplementary material, figure S3). The full system of nonlinear ordinary differential equations (ODEs) is given by the following equations:


$$\frac{\partial S\left(t\right)}{\partial t}=p_{S}-d_{S}\;S\left(t\right)-\;\beta_{0}\left(1-B\left(t\right)\right)S\left(t\right)V\left(t\right),$$

$$\frac{\partial I\left(t\right)}{\partial t}=\beta_{0\;}\left(1-B\left(t\right)\right)S\left(t\right)V\left(t\right)-d_{I}I\left(t\right),$$

$$\frac{\partial V\left(t\right)}{\partial t}=p_{V}I\left(t\right)-d_{V}V\left(t\right)-\;\beta_{0\;}\left(1-B\left(t\right)\right)S\left(t\right)V\left(t\right)$$

and


$$\frac{\partial B\left(t\right)}{\partial t}=p_{B}\left(1-B\left(t\right)\right)V\left(t\right),$$

where *S*(0) = *S*_0_, *I*(0) = *I*_0_, *V*(0) = *V*_0_ and *B*(0) = 0 are the given initial values (table 1). Susceptible cells are constantly produced at the rate *p*_*S*_ and die with the natural death rate *d*_S_, where *p*_*S*_ = *d*_*S*_*S*_0_. When in contact with a free virus, susceptible cells get infected according to an overall infectivity rate *β*_0_(1 − *B*(*t*)), where *β*_0_ is the infectivity rate. The term (1 − *B*(*t*)) indicates the proportion of remaining successful infections not prevented by the immune response. The successfully infected cells enter the infected cell population and are eliminated with the rate *d*_I_. Infected cells constantly produce and release free virus at the rate *p*_*V*_, which is cleared at the rate *d*_*V*_. Free virus leading to successfully infected cells are lost from this population according to the overall infectivity rate *β*_0_(1 − *B*(*t*)). This type of approach allows for a global description of the immune response–virus interaction in tractable mathematical terms by maintaining a biological interpretation. We assumed the individual to not have been exposed to SARS-CoV-2 prior to this infection, hence, *B*(0) = 0. Upon infection, *B* increases with increasing viral load at the rate *p_B_* and is limited to a maximal relative strength of 1, such that *B*(t) ∈ [0, 1]. We considered the activation of the immune response to lead to a reduced overall infectivity rate *β_0_*(1−*B*(*t*)), and hence, to fewer successful infections of susceptible cells [16,32]. According to how the immune response *B* impacts the overall infectivity rate *β*_0_(1*−B*) in the model, an upper bound of *B* is necessary to avoid negative overall infectivity rates and, hence, to ensure biological meaningfulness. As this model is used to describe the short-term acute infection dynamics only, we ignore a decrease in the immune response to long-term post-acute infection levels. For more details on the full model accounting also for post-acute infection dynamics, see [18]. Overall, the model is described by four populations, *S*, *I*, *V* and *B*, and seven parameters, *p_S_*, *d_S_*, *β*_0_, *d_I_*, *p_V_*, *d_V_* and *p_B_*, where all parameters are assumed to be positive for biological interpretability (table 1).

### 4.6. Within-host SARS-CoV-2 model with antiviral treatment remdesivir

Remdesivir inhibits viral replication [37]. In this model, we assumed the viral production rate *p*_V_ to be compromised by a factor of 1 − α, where α is the proportion of reduced viral production due to treatment and is termed effectiveness. Here, α ∈ [0, 1] (electronic supplementary material, figure S3). If α = 0, then remdesivir treatment is completely ineffective, while α = 1 signifies a complete inhibition of viral production. We adjusted the third equation of the previous ODE system accordingly to


$$\frac{\partial V\left(t\right)}{\partial t}=\left(1-\alpha \right)p_{V}I\left(t\right)-d_{V}V\left(t\right)-\;\beta_{0\;}\left(1-B\left(t\right)\right)S\left(t\right)V\left(t\right).$$

Remdesivir treatment was started on the day of hospitalization and was administered for five consecutive days. We assumed remdesivir treatment to be effective immediately and to be fully cleared after the last administration. After concluding the treatment, viral production is assumed to resume at its original rate *p*_*V*_.

### 4.7. Parametrization of the within-host SARS-CoV-2 model without and with treatment

To describe the individual SARS-CoV-2 infection dynamics, we parametrized the model as shown in table 1.

Initial values and the rate constants for five model parameters were taken from the literature and assumed to be the same across individuals ([18] for more details). To account for the heterogeneity between individuals, we estimated the viral production rate *p*_*V*_ and the activation rate of the immune response *p*_*B*_ at an individual-specific level. Additionally, we estimated the incubation period, i.e. the duration from infection to reported symptom onset, *t*_sympt_ per individual. The upper and lower boundaries of *p*_*V*_ were determined by the range of estimated individual-specific parameters from Ke *et al*. [16]. The lower and upper boundaries of *p*_*B*_ are less interpretable and, hence, were assumed to cover a broad range of values, while the boundaries for *t*_sympt_ were set from 2 up to 14 days [24]. For the model with treatment, we also estimated the individual-specific effectiveness of remdesivir, which according to its definition is bounded between 0 and 1.

### 4.8. Parameter estimation

To describe the transformed CT values using the model, we formally redefined population *V* as the virus in the sample and, similarly, *p*_*V*_ as the viral production rate times the proportion of sampled virus [16]. Accordingly, we also redefined *d*_*V*_, *p*_*B*_ and *β*_0_ times a factor reflecting the sampling process. To directly compare CT values and simulated viral loads, we made use of the CT value-to-viral load calibration determined by Ke *et al*. [16], given by


$$CT=\;-\;\frac{log10\;V-11.35}{0.25}.$$

Experimental data such as the measurements of CT values are noise corrupt. We took this measurement noise into account in the model, by assuming an additive Gaussian measurement noise distribution. The log-likelihood for the Gaussian noise model for individual *i* time point *k* and replicate *j* with measured CT values ${\overset{-}{y}}_{ji}^{k}$ is given by


$$logL\left(\theta_{i}\right)=-\frac{1}{2}\sum_{j}\sum_{k}\log\left(2\;\pi \;\sigma_{i}^{2}\right)+\;\frac{{\left({\overset{-}{y}}_{ji}^{k}-y_{i}\left(t_{k},\theta_{i}\right)\right)}^{2}}{\sigma_{i}^{2}}.$$

To account for the individual-specific measurement noise, we inferred an additional parameter σ determining the spread of the Gaussian noise model. The lower and upper bounds of σ were set to [10^−2^, 10]. In total, we estimated four parameters ($\theta_{i}$= {*p*_*V*_, *p*_*B*_, *t*_sympt_, σ}) for the untreated individuals (figure 2) and five parameters ($\theta_{i}$= {*p*_*V*_, *p*_*B*_, *t*_sympt_, α, σ}) for the treated individuals (figure 4). We then performed multi-start maximum likelihood optimization of the negative log-likelihood in the log_10_ parameter space for numerical reasons [38], initiating the optimization runs from 100 different Latin-hypercube-sampled starting points and maximizing over the CT values per individual. We used the MATLAB functions *fmincon* for optimization [39] and *ode45* for solving the ODEs [40].

### 4.9. Model selection

For the 19 treated individuals, we fitted the model without and with treatment to the viral loads (figure 4). To quantitatively compare the performance of both models and to determine the relative importance of treatment in the model, we used the Bayesian information criterion (BIC),


BIC=ln⁡(n)k−2logL,

where *n* is the number of data points, *k* is the number of estimated parameters and log*L* is the log-likelihood value for the maximum likelihood estimate of the model parameters. The BIC rewards high likelihood values and penalizes additional model parameters. Hence, low BIC values are preferable. We considered a BIC difference of more than 10 between the two models to be sufficient evidence to reject the model with the higher BIC [41] (electronic supplementary material, table S3).

### 4.10. Sensitivity analysis

For three randomly selected, representative individuals of each of the four sub-cohorts, we computed the profile likelihoods (electronic supplementary material, figures S4E, S4F, S6F and S6G). For each of the estimated model parameters (three for untreated individuals and four for treated individuals), we determined 99 evenly spaced values between 0.9 and 1.1 of the optimized value for a given model parameter and re-evaluated the negative log-likelihood for the given new parameter set.

### 4.11. Statistical comparison of timing, viral load and immune response features and model parameters

We compared the distributions and medians of a total of 24, 29 and 29 timing, viral load and immune response features and estimated model parameters between hospitalized and non-hospitalized untreated individuals (figure 3), between survived and deceased treated individuals (figure 5), and between treated individuals responding and not-responding to treatment (figure 6). For more detailed information see electronic supplementary material, table S2. We used the two-sample Kolmogorov–Smirnov and Mood’s median tests as provided by the MATLAB functions *kstest2* [42] and *mediantest* [43] to compare the distributions and medians of the given features, respectively. Both methods identify the (almost) same set of features as statistically significantly different between the tested sub-cohorts. We, hence, report the *p*-values corresponding to the two-sample Kolmogorov–Smirnov test in the main text only. As we were interested in every single feature separately, we did not correct for multiple testing.

## Data Availability

The MATLAB code corresponding to this manuscript can be found via a permanent repository on Zenodo [44]. Some restrictions apply to the clinical and epidemiological data and are available to interested researchers upon request to the Research Ethics Committee of the University of Thessaly (contact: ehde@uth.gr). Supplementary material is available online [45].

## References

[1] Beigel JH *et al*. 2020 Remdesivir for the treatment of Covid-19 – final report. N. Engl. J. Med. **383**, 1813–1826. (10.1056/NEJMoa2007764) 32445440 PMC7262788

[2] Tanni SE, Silvinato A, Floriano I, Bacha HA, Barbosa AN, Bernardo WM. 2022 Use of remdesivir in patients with COVID-19: a systematic review and meta-analysis. J. Bras. Pneumol. **48**, e20210393. (10.36416/1806-3756/e20210393) 35137874 PMC8836613

[3] Spinner CD *et al*. 2020 Effect of remdesivir vs standard care on clinical status at 11 days in patients with moderate COVID-19. JAMA **324**, 1048. (10.1001/jama.2020.16349) 32821939 PMC7442954

[4] Wang Y *et al*. 2020 Remdesivir in adults with severe COVID-19: a randomised, double-blind, placebo-controlled, multicentre trial. The Lancet **395**, 1569–1578. (10.1016/S0140-6736(20)31022-9) PMC7190303 32423584

[5] Pan H *et al*, WHO Solidarity Trial Consortium. 2021 Repurposed antiviral drugs for Covid-19 – interim WHO Solidarity Trial results. N. Engl. J. Med. **384**, 497–511. (10.1056/NEJMoa2023184) 33264556 PMC7727327

[6] Ader F, Bouscambert-Duchamp M, Hites M, Peiffer-Smadja N, Mentré F, Burdet C, DisCoVeRy Study Group. 2022 Final results of the DisCoVeRy trial of remdesivir for patients admitted to hospital with COVID-19. Lancet Infect. Dis. **22**, 764–765. (10.1016/S1473-3099(22)00295-X) 35643099 PMC9132543

[7] Barratt-Due A *et al*. 2021 Evaluation of the Effects of Remdesivir and hydroxychloroquine on viral clearance in COVID-19 : a randomized trial. Ann. Intern. Med. **174**, 1261–1269. (10.7326/M21-0653) 34251903 PMC8279143

[8] Wölfel R *et al*. 2020 Virological assessment of hospitalized patients with COVID-2019. Nature **581**, 465–469. (10.1038/s41586-020-2196-x) 32235945

[9] Néant N *et al*. 2021 Modeling SARS-CoV-2 viral kinetics and association with mortality in hospitalized patients from the French COVID cohort. Proc. Natl Acad. Sci. USA **118**, e2017962118. (10.1073/pnas.2017962118) 33536313 PMC7929555

[10] Owens K, Esmaeili S, Schiffer JT. 2024 Heterogeneous SARS-CoV-2 kinetics due to variable timing and intensity of immune responses. JCI Insight **9**, e176286. (10.1172/jci.insight.176286) 38573774 PMC11141931

[11] Biancofiore A, Mirijello A, Puteo MA, Di Viesti MP, Labonia M, Copetti M, De Cosmo S, Lombardi R, CSS-COVID-19 Group. 2022 Remdesivir significantly reduces SARS-CoV-2 viral load on nasopharyngeal swabs in hospitalized patients with COVID-19: a retrospective case-control study. J. Med. Virol. **94**, 2284–2289. (10.1002/jmv.27598) 35043405 PMC9015337

[12] Campogiani L *et al*. 2023 Remdesivir influence on SARS-CoV-2 RNA viral load kinetics in nasopharyngeal swab specimens of COVID-19 hospitalized patients: a real-life experience. Microorganisms **11**, 312. (10.3390/microorganisms11020312) 36838277 PMC9959460

[13] Goldberg E, Ben Zvi H, Sheena L, Sofer S, Krause I, Sklan EH, Shlomai A. 2021 A real-life setting evaluation of the effect of remdesivir on viral load in COVID-19 patients admitted to a large tertiary centre in Israel. Clin. Microbiol. Infect. **27**, 917.(10.1016/j.cmi.2021.02.029) PMC7939997 33705849

[14] Goyal A, Duke ER, Cardozo-Ojeda EF, Schiffer JT. 2022 Modeling explains prolonged SARS-CoV-2 nasal shedding relative to lung shedding in remdesivir-treated rhesus macaques. i. Sci. **25**, 104448. (10.1016/j.isci.2022.104448) PMC9130309 35634576

[15] Challenger JD, Foo CY, Wu Y, Yan AWC, Marjaneh MM, Liew F, Thwaites RS, Okell LC, Cunnington AJ. 2022 Modelling upper respiratory viral load dynamics of SARS-CoV-2. BMC Med. **20**, 25. (10.1186/s12916-021-02220-0) 35022051 PMC8755404

[16] Ke R *et al*. 2022 Daily longitudinal sampling of SARS-CoV-2 infection reveals substantial heterogeneity in infectiousness. Nat. Microbiol. **7**, 640–652. (10.1038/s41564-022-01105-z) 35484231 PMC9084242

[17] Perelson AS, Ke R. 2021 Mechanistic modeling of SARS-CoV-2 and other infectious diseases and the effects of therapeutics. Clin. Pharmacol. Ther. **109**, 829–840. (10.1002/cpt.2160) 33410134 PMC8142935

[18] Schuh L, Markov PV, Veliov VM, Stilianakis NI. 2023 A mathematical model for the within-host (re)infection dynamics of SARS-CoV-2. Math. Biosci. **371**, 109178. (10.1016/j.mbs.2024.109178)38490360

[19] Lingas G *et al*. 2022 Effect of remdesivir on viral dynamics in COVID-19 hospitalized patients: a modelling analysis of the randomized, controlled, open-label DisCoVeRy trial. J. Antimicrob. Chemother. **77**, 1404–1412. (10.1093/jac/dkac048) 35233617 PMC9383489

[20] Phan T *et al*. 2023 Modeling the emergence of viral resistance for SARS-CoV-2 during treatment with an anti-spike monoclonal antibody. bioRxiv. (10.1101/2023.09.14.557679) PMC11060554 38635853

[21] Ruysseveldt E, Martens K, Steelant B. 2021 Airway basal cells, protectors of epithelial walls in health and respiratory diseases. Front. Allergy **2**, 787128. (10.3389/falgy.2021.787128) 35387001 PMC8974818

[22] Szablewski CM *et al*. 2020 SARS-CoV-2 transmission and infection among attendees of an overnight camp — Georgia, June 2020. MMWR Morb. Mortal. Wkly. Rep. **69**, 1023–1025. (10.15585/mmwr.mm6931e1) 32759921 PMC7454898

[23] Ferretti L *et al*. 2020 The timing of COVID-19 transmission. SSRN Journal (10.2139/ssrn.3716879)

[24] Elias C, Sekri A, Leblanc P, Cucherat M, Vanhems P. 2021 The incubation period of COVID-19: a meta-analysis. Int. J. Infect. Dis. **104**, 708–710. (10.1016/j.ijid.2021.01.069) 33548553 PMC7857041

[25] Zitzmann C, Ke R, Ribeiro RM, Perelson AS. 2024 How robust are estimates of key parameters in standard viral dynamic models? PLoS Comput. Biol. **20**, e1011437. (10.1371/journal.pcbi.1011437) 38626190 PMC11051641

[26] Rhee C, Kanjilal S, Baker M, Klompas M. 2021 Duration of severe acute respiratory syndrome Coronavirus 2 (SARS-CoV-2) infectivity: when is it safe to discontinue isolation? Clin. Infect. Dis. **72**, 1467–1474. (10.1093/cid/ciaa1249) 33029620 PMC7499497

[27] Centers for Disease Control and Prevention. 2023 Ending isolation and precautions for people with COVID-19: interim guidance. See https://archive.cdc.gov/#/details?q=https://www.cdc.gov/coronavirus/2019-ncov/hcp/duration-isolation.html&start=0&rows=10&url=https://www.cdc.gov/coronavirus/2019-ncov/hcp/duration-isolation.html (accessed 14 November 2024).

[28] Svanberg R *et al*. 2022 Early stimulated immune responses predict clinical disease severity in hospitalized COVID-19 patients. Commun. Med. **2**, 114. (10.1038/s43856-022-00178-5) 36101705 PMC9466310

[29] Adéoti OM, Agbla S, Diop A, Glèlè Kakaï R. 2025 Nonlinear mixed models and related approaches in infectious disease modeling: a systematic and critical review. Infect. Dis. Model. **10**, 110–128. (10.1016/j.idm.2024.09.001) 39376223 PMC11456789

[30] Wu Y, Kang L, Guo Z, Liu J, Liu M, Liang W. 2022 Incubation period of COVID-19 caused by unique SARS-CoV-2 strains: a systematic review and meta-analysis. JAMA Netw. Open **5**, e2228008. (10.1001/jamanetworkopen.2022.28008) 35994285 PMC9396366

[31] Ke R, Zitzmann C, Ho DD, Ribeiro RM, Perelson AS. 2021 In vivo kinetics of SARS-CoV-2 infection and its relationship with a person’s infectiousness. Proc. Natl Acad. Sci. USA **118**, e2111477118. (10.1073/pnas.2111477118) 34857628 PMC8670484

[32] Marc A *et al*. 2023 Impact of variants of concern on SARS-CoV-2 viral dynamics in non-human primates. PLoS Comput. Biol. **19**, e1010721. (10.1371/journal.pcbi.1010721) 37556476 PMC10441782

[33] Ranard BL, Chow CC, Megjhani M, Asgari S, Park S, Vodovotz Y. 2023 A mathematical model of SARS-CoV-2 immunity predicts paxlovid rebound. J. Med. Virol. **95**, e28854. (10.1002/jmv.28854) 37287404 PMC10264150

[34] Perelson AS, Ribeiro RM, Phan T. 2023 An explanation for SARS-CoV-2 rebound after Paxlovid treatment. medRxiv 2023.05.30.23290747. (10.1101/2023.05.30.23290747)

[35] The MathWorks Inc. binornd: random numbers from binomial distribution*.* See https://it.mathworks.com/help/stats/binornd.html.

[36] cdf. 2024 Cumulative distribution function - MATLAB - MathWorks Italia. See https://it.mathworks.com/help/stats/prob.normaldistribution.cdf.html (accessed 27 September 2024).

[37] Kokic G *et al*. 2021 Mechanism of SARS-CoV-2 polymerase stalling by remdesivir. Nat. Commun. **12**, 279. (10.1038/s41467-020-20542-0) 33436624 PMC7804290

[38] Hass H, Loos C, Raimúndez-Álvarez E, Timmer J, Hasenauer J, Kreutz C. 2019 Benchmark problems for dynamic modeling of intracellular processes. Bioinformatics **35**, 3073–3082. (10.1093/bioinformatics/btz020) 30624608 PMC6735869

[39] The MathWorks Inc. fmincon - find minimum of constrained nonlinear multivariable function. See https://it.mathworks.com/help/optim/ug/fmincon.html.

[40] The MathWorks Inc. ode45 - solve nonstiff differential equations — medium order method. See https://it.mathworks.com/help/matlab/ref/ode45.html.

[41] Kass RE, Raftery AE. 1995 Bayes factors. J. Am. Stat. Assoc. **90**, 773–795. (10.1080/01621459.1995.10476572)

[42] MathWorks. kstest2 - Two-sample Kolmogorov-Smirnov test. See https://it.mathworks.com/help/stats/kstest2.html.

[43] Keine C. Mood’s median test. See https://it.mathworks.com/matlabcentral/fileexchange/70081-moods-median-test.

[44] lea-schuh. 2024 lea-schuh/COVID_treat: v1.0.0 (v1.0.0). Zenodo (10.5281/zenodo.13905096)

[45] Schuh L, Markov PV, Voulgaridi I, Bogogiannidou Z, Mouchtouri VA, Hadjichristodoulou C *et al*. 2024 Supplementary material from: Within-host dynamics of antiviral treatment with remdesivir for SARS-CoV-2 infection. Figshare. (10.1101/2024.05.31.24308284)39592013

